# Practical Observations on the Pathology and Treatment of Certain Diseases of the Skin, &c.

**Published:** 1847-07-01

**Authors:** 


Practical Observations on the Pathology and Treatment of certain Diseases of the Skin, generally pronounced intractable. By *Thomas Hunt.* Octavo, pp. 152. London, Churchill, 1847.

If we are to believe our author, Arsenic, administered according to his method, is all but an infallible remedy in the worst case of Lichen, Prurigo, Lepra, Psoriasis, Pityriasis, chronic Urticaria, Purpura, Ecthyma, Eczema, Acne, and Sycosis, and also in Lupus and in non-congenital Nævus. All the varieties of these diseases, if not of a syphilitic origin, or not associated with feverishness or other derangement of the constitutional health, are declared by him to be under the dominion of "one antidote, which, under proper regulations, acts with such uniform efficiency as to leave nothing to be desired. That remedy is *arsenic.*"

Let us first see what these "proper regulations" are. In place of the usual method of giving arsenic in small doses at first, and gradually increasing them until either some impression is made upon the existing disease, or certain toxical symptoms are induced, Mr. Hunt contends that it is much safer and better to begin with full or tolerably large doses at first, and reduce them by degrees as their *effects* become palpable. The most characteristic of these effects is a pricking sensation in the tarsi, and slight inflammation of the conjunctiva. On this subject, the author makes the following remarks.

"*At this crisis* (the supervention of these ophthalmic symptoms) *the disease is brought under arrest, and generally from this period appears to be shorn of its strength.* The return of healthy action in the cutaneous vessels often becomes visible, and is sensibly felt by the patient, on the very day on which the eyes become suffused with tears. The dose may now be reduced, and in some cases a very small dose taken with exact regularity will suffice to keep the eyes tender and the skin healing, until at length even the disposition to disease appears to die away under the influence of the poison. And as in the exhibition of mercury we are content with making the gums sore without distressing the bowels, so in administering arsenic we should never allow the mucous membranes to suffer: for supervening gastritis, and even cholicky pains, most clearly contra-indicate its use. Fortunately a slight degree of conjunctivitis, in about forty-nine cases out of fifty takes precedence of the more grave affections which indicate an over-dose. Both the safety of the patient and the prospect of his recovery will much depend upon the vigilance with which a knowledge of this fact inspires the surgeon. Ignorance of the existence of this safety-valve has caused many a cautious practitioner to repudiate the medicine altogether; and an acquaintance with this important sign would, doubtless, on the other hand, have checked the temerity which, in its results, has attainted with unmerited suspicion the reputation of a valuable remedy." P. 13.

Arsenic, when taken for a considerable length of time, is said to produce in some cases a discolouration of the skin:

"The trunk of the patient first, and subsequently all those parts of the body which are by the dress protected from the access of light and air, become covered with a dirt-brown, dingy, unwashed appearance, which, under a lens, reveals a delicate desquamation of the dermis, and is, in fact, a faint form of pityriasis. This may be considered as a secondary form of arsenicalization; for I have observed that when the primary dose is diminished on the appearance of conjunctivitis, the eye-lids may be allowed to get well, yet if the patient's skin be kept *brown*, the disease will vanish just as rapidly as though the conjunctiva were kept sore. The first and larger dose appears to knock the disease on the head, (so to speak,) and to exhaust its energy. Its less malign, or secondary form, being subjugated by the secondary or pityriatic action of arsenic." P. 15.

In old chronic cases of cutaneous affections, "the arsenical course should be protracted (in reduced doses) for about as many *months* after the final disappearance of the disease, as it had existed *years* before. This will prove the best security against a relapse, and will generally succeed in preventing it."

After narrating the details of upwards of 40 successful cases—we hear indeed of no unsuccessful ones—of various forms of skin-disease, including four of *lupus exedens* and one of *nævus*, in which a cure was effected by the use of arsenic, Mr. Hunt favours us with some general observations. From these we select the following passage as a fair specimen of his mode of handling his subject.

"It is now many years since I resolved to try what could be accomplished by arsenic in the treatment of the more unmanageable disorders of the skin, and the result has filled me with astonishment and delight. Whenever I have had a fair trial and a fitting case, I can truly say that for many years arsenic has never once failed me. In the few cases which have not done well, either there was irreparable organic disease existing, or else the irregular habits or whims of the patient, or some accidental interruption to the course, sufficiently accounted for the failure. It is possible that in *some* cases related as cured, relapses may have occurred unknown to me. I can only relate facts as far as they have come to my knowledge, and leave others to draw their conclusions. In the majority of the cases, I have ascertained that the patient has for a greater or lesser period, after the termination of the course, enjoyed immunity from the disease which had tortured him for years.

"Of other alteratives, as respects their efficiency in cutaneous disease, I know but little except from report. Of their very general *inefficiency* I had painful experience in the early part of my practice. I have long abjured medicated baths, and external applications generally; and, excepting where the antiphlogistic regimen was required, *I have placed no restriction whatever upon the diet of my patients.* I never could understand the principles on which the dietetic system of treating disease, is founded. If the appetite is not depraved by gross intemperance, I look upon it to be the only safe indication of the proper quality and quantity of food required in a given case, both in health and disease. I do not say it may never prove false: and I will promise never to trust it, if any man will supply me with a more intelligent or more philosophical guide. Neither local treatment nor diet therefore, (with the above exception,) have had any influence in determining the results of my experiments. But in order to submit the agency of arsenic to a still more severe test, I have in almost every protracted case, interrupted the course again and again, and have found to my unspeakable satisfaction that, in every case unshackled by complications, I could as readily check the disease, and allow it to advance at pleasure, as the engineer can control the progress of his locomotive. The power of the medicine in these cases is therefore established beyond the reach of doubt or cavil. And its safety is not less demonstrable. By discovering the efficacy of its continued use in small and *decreasing* doses, and thus securing for the medicine an innocuous operation, I trust I have removed the only valid objection to its use, namely, its dangerous properties. And now, with the exception of its name, and the horrors associated with the idea of a poison, one can scarcely conceive a remedy less objectionable than arsenic. Sarsaparilla, besides being nearly useless, is expensive; the preparations of cantharides irritate the urinary organs; iodine, besides that it is 'no respecter of tissues,' is a nauseous medicine, and few patients could be prevailed upon to take it, were it safe to do so, for a lengthened period. But none of these objections apply to arsenic. A medicine which, besides being almost or quite certain in its operation, is safe, cheap, tasteless, and elegant—which may be taken at meal-times through a whole life, if necessary, without creating disgust or nausea,—which interferes (in curative doses) with no healthy function,—which gives no pain, and inflicts no inconvenience—has surely recommendations which are not easily surpassed." P. 134.

We must close our notice of Mr. Hunt's volume with the same words of hesitating belief with which we began it. His style of writing is not likely to beget much confidence in his statements, among those of his professional brethren whose good opinion is most worth having. The practice of authors publishing patients' eulogistic and recommendatory letters cannot be too much censured. It always savours of the advertising school, to which too many of the medical authors in the present day give countenance by their own practice.

